# Key radioresistance regulation models and marker genes identified by integrated transcriptome analysis in nasopharyngeal carcinoma

**DOI:** 10.1002/cam4.4228

**Published:** 2021-08-25

**Authors:** Zhuang Sun, Xiaohui Wang, Jingyun Wang, Jing Wang, Xiao Liu, Runda Huang, Chunyan Chen, Meiling Deng, Hanyu Wang, Fei Han

**Affiliations:** ^1^ Department of Radiation Oncology Sun Yat‐sen University Cancer Center Guangzhou People’s Republic of China; ^2^ State Key Laboratory of Oncology in South China Guangzhou People’s Republic of China; ^3^ Collaborative Innovation Center for Cancer Medicine Guangzhou People’s Republic of China; ^4^ Guangdong Key Laboratory of Nasopharyngeal Carcinoma Diagnosis and Therapy Guangzhou People’s Republic of China; ^5^ GFK biotec.Shanghai Inc

**Keywords:** nasopharyngeal carcinoma, radioresistance, radiosensitivity, RNA sequencing

## Abstract

Nasopharyngeal carcinoma (NPC) is a malignancy that is endemic to China and Southeast Asia. Radiotherapy is the usual treatment, however, radioresistance remains a major reason for failure. This study aimed to find key radioresistance regulation models and marker genes of NPC and clarify the mechanism of NPC radioresistance by RNA sequencing and bioinformatics analysis of the differences in gene expression profiles between radioresistant and radiosensitive NPC tissues. A total of 21 NPC biopsy specimens with different radiosensitivity were analyzed by RNA sequencing. Differentially expressed genes in RNA sequencing data were identified using R software. The differentially expressed gene data derived from RNA sequencing as well as prior knowledge in the form of pathway databases were integrated to find sub‐networks of related genes. The data of RNA sequencing with the GSE48501 data from the GEO database were combined to further search for more reliable genes associated with radioresistance of NPC. Survival analyses using the Kaplan–Meier method based on the expression of the genes were conducted to facilitate the understanding of the clinical significance of the differentially expressed genes. RT‐qPCR was performed to validate the expression levels of the differentially expressed genes. We identified 1182 differentially expressed genes between radioresistant and radiosensitive NPC tissue samples. Compared to the radiosensitive group, 22 genes were significantly upregulated and 1160 genes were downregulated in the radioresistant group. In addition, 10 major NPC radiation resistance network models were identified through integration analysis with known NPC radiation resistance‐associated genes and mechanisms. Furthermore, we identified three core genes, DOCK4, MCM9, and POPDC3 among 12 common downregulated genes in the two datasets, which were validated by RT‐qPCR. The findings of this study provide new clues for clarifying the mechanism of NPC radioresistance, and further experimental studies of these core genes are warranted.

## Graphical abstract

In this work, we identified several key radioresistance regulation models and marker genes of nasopharyngeal carcinoma (NPC) and partly demonstrated the mechanism of NPC radioresistance by RNA sequencing and bioinformatics analysis of the differences in gene expression profiles between radioresistant and radiosensitive NPC tissues.

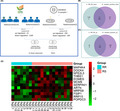

## 1 INTRODUCTION

Nasopharyngeal carcinoma (NPC) is an epithelial head and neck tumor with marked geographical variation in distribution. It is mostly prevalent in China and Southeast Asia.^1^ According to the 2018 global cancer statistics, there were about 129 079 new cases of NPC in 2018, accounting for 0.7% of all new cancer patients.^2^ Intensity‐modulated radiotherapy (IMRT) is the standard treatment method for patients with non‐metastatic NPC, which enables good tumor control.^3^, ^4^ However, 10%–20% of NPC patients develop recurrence after radiotherapy due to radioresistance.^5^ Notably, the prognosis of patients with recurrent NPC is still very poor.^6^, ^7^ Therefore, it is imperative to clarify the molecular mechanism underlying the radioresistance of NPC.

Previous studies have found some molecules and biological processes relevant to the radioresistance of NPC by analyzing radioresistant NPC cells and radiosensitive NPC cells.^8^, ^9^, ^10^, ^11^ For example, Chang et al.^8^ compared two radioresistant NPC cell lines with their corresponding parental cell lines by cDNA microarray and found that seven genes, including gp96/hsp90b1 and GDF15, were associated with the radioresistance of NPC. In addition, Li et al.^9^ identified 15 differentially expressed miRNAs and 372 differential mRNAs associated with NPC radioresistance by comparing radioresistant NPC CNE2‐IR cells with radiosensitive NPC CNE2 cells. However, to date only limited studies have focused on the analysis of the difference between radioresistant and radiosensitive clinical NPC tissues.

Therefore, in this study, we exploited RNA sequencing technology to explore the differences in gene expression profiles between radioresistant and radiosensitive NPC biopsy specimens. Together with known genes and mechanisms involved in the radioresistance of NPC, we set out to define the major radioresistance network models through dataset integration and to make inferences on the key genes. Furthermore, we combined the RNA sequencing results with the existing data in the Gene Expression Omnibus (GEO) database to filter out reliable molecular markers and investigate the impact of these markers on the survival rates of patients. The major regulation models described in this study provide new clues for elucidating the mechanism of radioresistance of NPC and may be used to develop new predictors of NPC radioresponse and patient prognosis.

## 2 MATERIALS AND METHODS

### 2.1 Patients and tissue samples

A total of 21 fresh frozen NPC biopsy specimens from the Sun Yat‐sen University Cancer Center were retrospectively collected from January 2013 to February 2017. All patients were pathologically diagnosed with non‐metastatic NPC, and tumor tissue samples were obtained before radiotherapy. The samples were immediately frozen in liquid nitrogen and stored. All patients received radiotherapy and platinum‐based chemotherapy. According to the response to radiotherapy, we divided the 21 NPC patients into two groups: a radiosensitive group with 14 patients and a radioresistant group with 7 patients. Here, radiosensitive patients were defined as NPC patients with no residual lesions after 6 weeks of radiotherapy and no recurrence within 5 years after radiotherapy, and radioresistant patients were defined as NPC patients with residual lesions after radiotherapy for more than 6 weeks or NPC patients with recurrence within 1 year after radiotherapy. All patients were re‐staged according to the 8th edition of the American Joint Committee on Cancer (AJCC) Staging Manual. Detailed clinical features of the 21 patients are shown in Table 1. This study was approved by the Ethical Review Committee of Sun Yat‐sen University Cancer Center, and informed consent was obtained from all patients.

**TABLE 1:** The clinical characteristics of nasopharyngeal carcinoma samples

	Radiosensitive (n=14)	Radioresistant (n=7)	*P* value
Age (mean ±SD)	45.57±7.76	40.6±8.32	0.190
Sex			0.006
Male	14	3	
Female	0	4	
T stage			0.374
T1	0	1	
T2	2	1	
T3	10	3	
T4	2	2	
N stage			0.677
N0	1	1	
N1	5	2	
N2	6	4	
N3	2	0	
TNM stage			0.866
II	1	1	
III	9	4	
IV	4	2	

### 2.2 Total RNA extraction

Total RNA was extracted using TRIzol reagent (Invitrogen, Carlsbad, CA, USA) according to the manufacturer's instructions. The purity and concentration of RNA were detected using a NanoDrop ND‐1000 spectrophotometer (NanoDrop, Wilmington, DE, USA). The integrity of RNA was verified using an Agilent 2100 Bioanalyzer (Agilent Technologies, USA) with an RNA integrity number (RIN) >7.0.

### 2.3 Library construction and RNA sequencing

Library construction and RNA sequencing were performed by LC Bio Inc. (Hangzhou, China). Approximately 5 μg of total RNA was extracted, and then ribosomal RNA (rRNA) was removed from the total RNA using the Ribo‐Zero™ rRNA Removal Kit (Illumina, San Diego, USA). The remaining RNA was fragmented and then synthesized into first‐strand cDNA using reverse transcriptase and random primers. Second‐strand cDNA synthesis was then performed using E. coli DNA polymerase I, RNase H, and dUTP. Next, the cDNA strands were end‐repaired and added an “A” base. They were then ligated to the indexed adapters which contained a “T” base overhang. After dUTP strand degradation by the treatment of the UDG enzyme, the cDNA products were amplified by polymerase chain reaction (PCR) for the formation of a library with a fragment size of 300 bp (±50 bp). Finally, paired‐end sequencing was performed using Illumina X Ten (LC Bio, China).

### 2.4 Principal component analysis

To clearly evaluate the similarities and differences between samples and determine whether samples were grouped correctly, principal component analysis (PCA) of two cohorts was conducted using the ellipse package in R software (version 3.5.0). PCA is a dimensionality reduction method that is used to reduce the dimensionality of large datasets, by transforming a large set of variables into a smaller one that still contains most of the information in the large set.^12^

### 2.5 Identification of differentially expressed genes

Clean sequencing reads were aligned with the index built from the human (hg37) genome, and the high‐quality reads were mapped to the reference by HISTA2 v2.1.0, and then the FPKM (fragments per kilobase per million) value of the genes and isoforms were calculated using StringTie v2.1.4 using a combination of Illumina and full‐length transcript‐based annotations.

Differentially expressed genes in RNA sequencing data were identified using the genefilter package in R software (version 3.5.0). Genes with a fold change greater than 1.5 (FC>1.5) and *P *< 0.05 between the radioresistant and radiosensitive groups were considered significant.

### 2.6 GO and KEGG pathway enrichment analyses

To understand the functional properties of the differentially expressed genes, Gene Ontology (GO, http://geneontology.org/) and Kyoto Encyclopedia of Genes and Genomes (KEGG, http://www.genome.jp/kegg/) pathway analyses were performed using the OmicsBean workbench (http://www.omicsbean.cn), an online multiple omics data analysis application. GO analysis was utilized to characterize genes and gene products in terms of cellular component (CC), biological process (BP), and molecular function (MF). KEGG pathway analysis was performed to further identify the pathways in which the differentially expressed genes underwent significant enrichment, thus predicting the potential functions of the differentially expressed genes. In addition, the pathway activation strength (PAS) prediction algorithm^13^ implemented in the omicsbean workbench was used to predict the effects of the processes identified by GO enrichment analysis. A positive value of PAS indicates the activation of a signaling pathway, while a negative value indicates the inhibition of a signaling pathway.^13^

### 2.7 Summary of the known genes and mechanisms associated with radioresistance

To summarize the influencing factors and regulatory mechanisms of tumor radiosensitivity and radioresistance that have been known, we retrieved relevant literature from January 1990 to October 2020 using PubMed with the keywords of “radiosensitivity,” “radiation sensitivity,” “radioresistance,” “radiation resistance,” and “cancer.” The representative genes and mechanisms retrieved are listed in Table 2.

**TABLE 2:** Known genes and mechanisms associated with radioresistance

Radioresistance‐related mechanisms	Radioresistance‐associated genes	Refs
Enhanced DNA damage repair	MRE11, RAD50, NBS1, ATM, ATR, RAD51, BRCA1, BRCA2, DNA‐PK, XRCC4, LIG4, H2AX, MDMX, MDM2, MDC1, 53BP1, TLK1, Rad9, ATF2, and SMC1	48, 49, 50, 51, 52, 53, 54, 55, 56, 57, 58, 59
Altered cell cycle	Chk1, Chk2, CDC25A, CDK2, CDC25C, CDK1, p21, p16, GADD45, NF‐kappa‐B, and FANCD2	60, 61, 62, 63, 64, 65, 66, 67, 68
Evasion of apoptosis	TP53, Bcl2, Bax, FAS, TNF, TRAIL, Livin, XIAP, CIAP1, CIAP2, Survivin, Smac, Caspase, RelB, CREB, and SAPK	69, 70, 71, 72, 73, 74
Hypoxia	HIF1	75, 76, 77
Angiogenesis	VEGF	^78^

### 2.8 Construction of “hub” sub‐network models for the radioresistant system

Through analyzing the relationships between the sequenced differential genes and the reported marker genes related to radiosensitivity, that is, including protein–protein interactions and pathways that genes collectively participate in the “hub” sub‐network models related to radiosensitivity were constructed. Specifically, the sub‐network models were generated by the Cytoscape web application,^14^ based on the information obtained from four levels of functional analysis: fold change of genes/proteins, protein–protein interactions, KEGG pathway enrichment, and biological process enrichment. The STRING database (search tool for the retrieval of interacting genes/proteins, http://string‐db.org)^15^ was used to analyze protein–protein interactions. Go and KEGG analyses were performed for pathway enrichment analysis.

### 2.9 Combination analyses of differentially expressed genes with GEO data

The microarray dataset GSE48501 based on the Affymetrix Human Genome U133 Plus 2.0 Array platform was downloaded from the GEO database (http://www. ncbi.nlm.nih.gov/geo). The dataset contained the mRNA expression profiles of two samples of radioresistant NPC CNE2‐IR cells and two samples of radiosensitive NPC CNE2 cells. NPC CNE2‐IR cells were derived from the poorly differentiated NPC cell line CNE2 by treating the cells with four rounds of a sublethal dose of radiation. Our RNA sequencing results were combined with the data from GSE48501 to identify overlapping differentially expressed genes. The potential functions of the overlapping differentially expressed genes were further analyzed.

### 2.10 RT‐qPCR validation for the expression of the differentially expressed genes

The expression of the differentially expressed genes was detected by reverse transcription‐quantitative polymerase chain reaction (RT‐qPCR). Specific primer sequences for the genes are shown in Table 3. Total RNA (2 µg) was reverse transcribed into cDNA using a Reverse Transcription Kit according to the manufacturer's protocol. Then, using cDNA as the template, qPCR was performed using qPCR Mix under the following reaction conditions: the initial denaturation step was 95℃ for 10 min, followed by 40 cycles of 95℃ for 10 seconds and 60℃ for 1 min. The internal reference gene was GAPDH, and the relative expression levels of the genes were calculated by the 2‐^ΔΔCt^ method.

**TABLE 3:** A list of primers used in this study

Gene	Primer Sequence (5’−3’)
DOCK4	F:ATTCCAGAGAGCCAGGAGGT
	R:TGACGTTCTCTCCACCCAGA
MCM9	F:AGGTTCTGGAGTTTGAGCGG
	R:ACAAGCCTGAGAGGCAAGTG
POPDC3	F:TGCACAACCTGGAAGCAAGA
	R:AGAAAACCCAACCCCAGCAA
GAPDH	F:GCATCCTGGGCTACACTGAG
	R:AAAGTGGTCGTTGAGGGCAA

### 2.11 Statistical analysis

Statistical analysis was conducted using R 3.0 (http://www.r‐project.org/). The results are presented as mean ±SD. For differential expression analysis, Student's *t*‐test between groups was used. The rates of overall survival (OS) and progression‐free survival (PFS) were calculated using the Kaplan–Meier method, and the differences in survival rates between patients with different gene expression levels were compared using the log‐rank test. *P* values <0.05 were considered statistically significant.

## 3 RESULTS

### 3.1 Differentially expressed genes between radiosensitive and radioresistant NPC tissues

**FIGURE 1:**
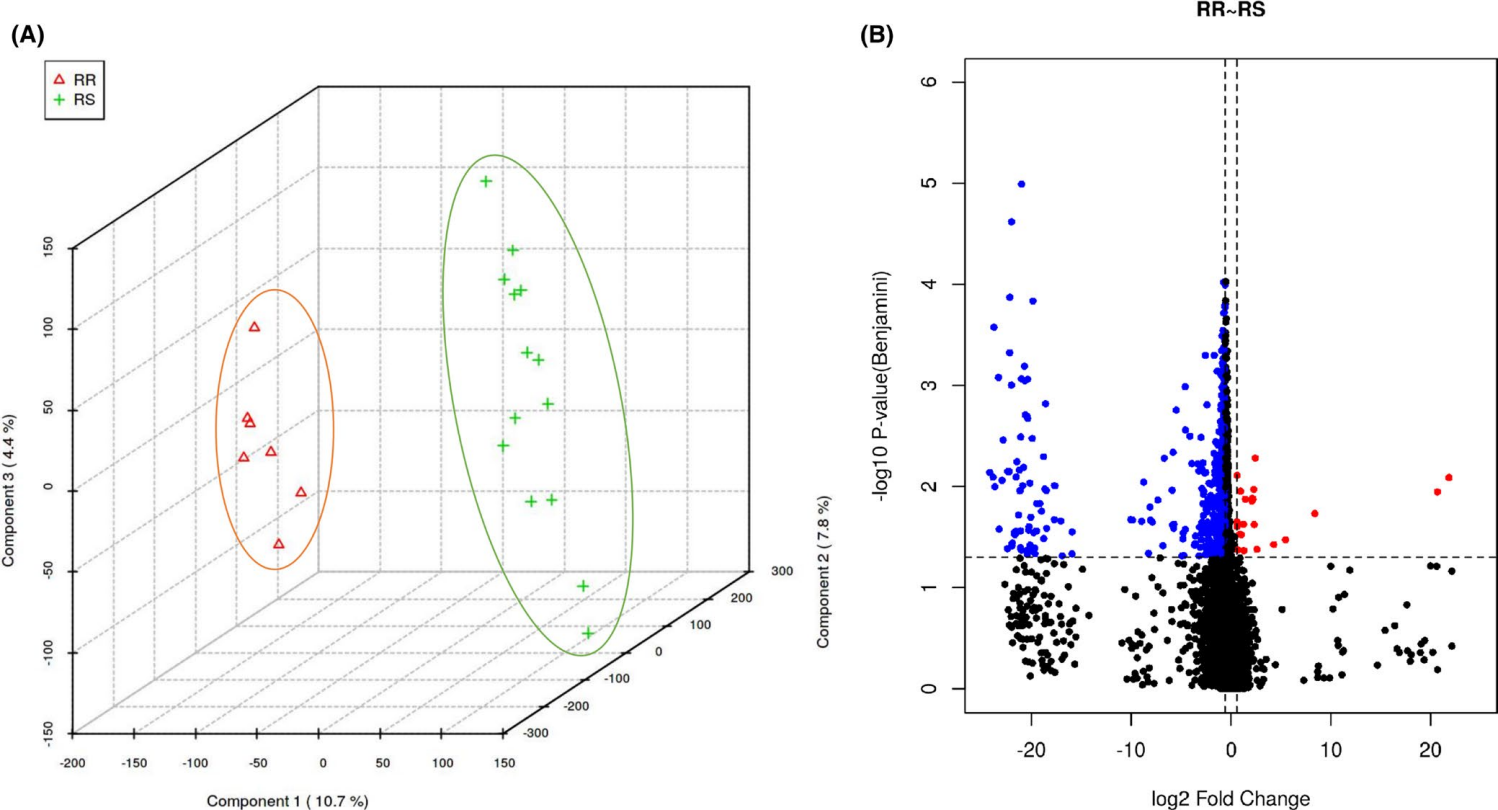
Identification and hierarchical clustering of differentially expressed genes. RR for radioresistant and RS for radiosensitive. (A) Principal component analysis (PCA) of two cohorts. (B) Volcano plot of differentially expressed genes between radioresistant and radiosensitive groups. The cutoff criteria were fold change >1.5 and *P *< 0.05. The red dots represent the upregulated genes and the blue dots signify the downregulated genes. The black dots indicate the genes with a fold change <1.5 and/or *P *> 0.05

In our dataset, the two independent cohorts (radioresistant group and radiosensitive group) can be clearly separated into two clusters with principal component analysis (PCA) (Figure 1A), suggesting that the radio impacts on the transcriptome exhibit expressional closeness within each group.

In total, we identified 1182 differentially expressed genes with filter criteria: fold change >1.5 and *P *< 0.05. Compared to the radiosensitive group, 22 genes were significantly upregulated and 1160 genes were downregulated in the radioresistant group (Figure 1B).

### 3.2 Gene ontology and KEGG analyses of the differentially expressed genes

**FIGURE 2:**
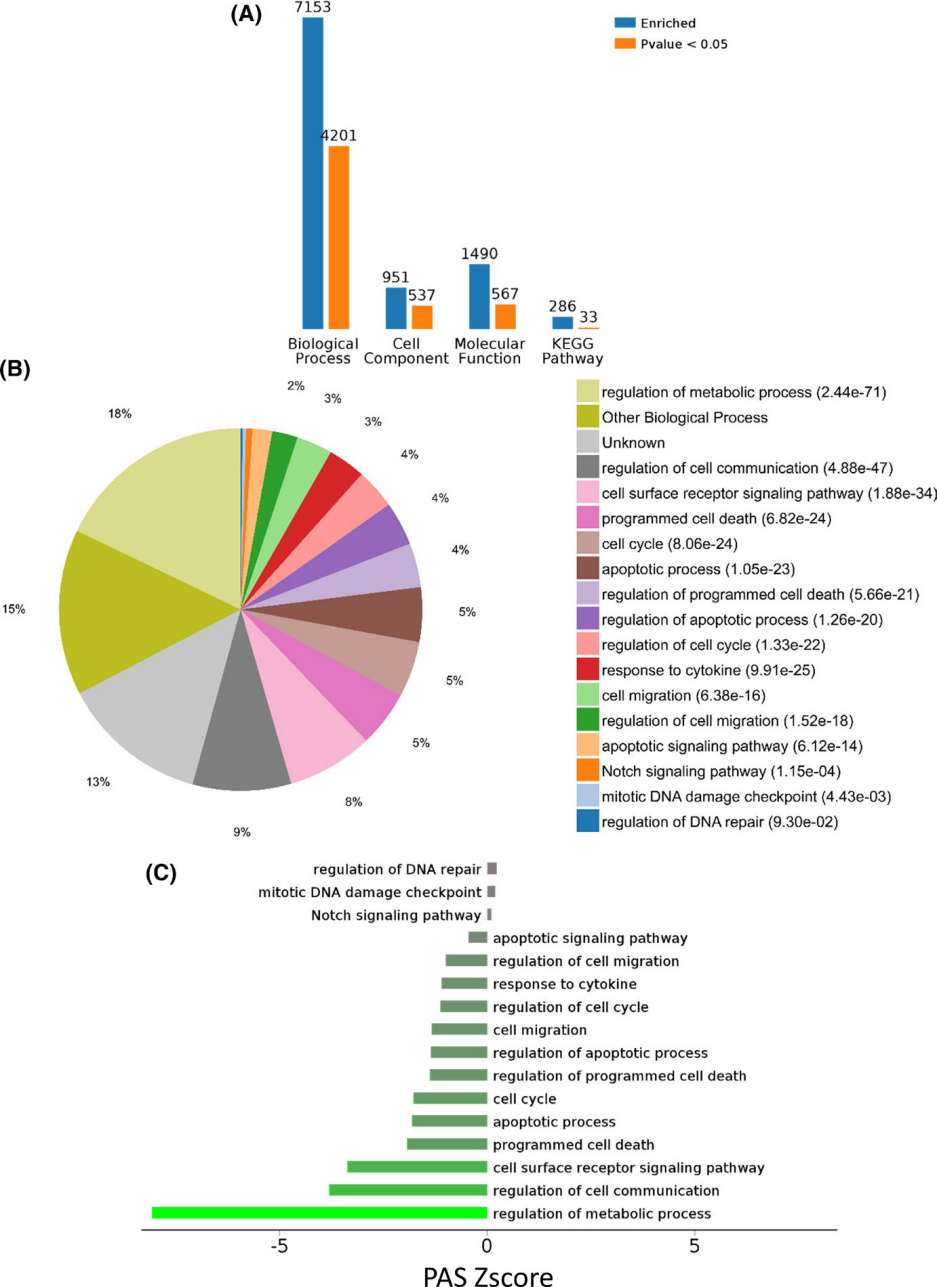
Enrichment analysis of the differentially expressed genes between the radioresistant and radiosensitive groups. (A) Gene ontology enrichment and KEGG pathway enrichment analysis of the differentially expressed genes. Blue and orange bars indicate enriched total terms and terms exhibiting statistical significance (*P *< 0.05) in biological process, cell component, molecular function, and KEGG pathway, respectively. (B) Significant (*P *< 0.05) biological processes enriched by Gene ontology analysis. (C) Predicted top activated and inhibited functional processes based on pathway activation strength (PAS) scores. Brown bars and green bars represent the degree of pathway activation or inhibition, respectively

Gene ontology analysis of the 1182 differentially expressed genes enriched in a total of 7153 BP, 951 CC, and 1490 MF terms (Figure 2A), among which 4201, 537, and 567 terms were significantly enriched (*P *< 0.05), respectively. KEGG pathway enrichment analysis revealed a total of 286 pathways (Figure 2A), among which 33 pathways met the *P *< 0.05 criteria.

Among the significantly enriched biological processes, regulation of the metabolic process, regulation of cell communication, apoptotic process, regulation of programmed cell death, and regulation of cell cycle were enriched, playing a leading role in radioresistant events. In addition, these differentially expressed genes were also significantly involved in cell migration, Notch signaling pathway, response to cytokine, mitotic DNA damage checkpoint, and regulation of DNA repair (Figure 2B).

Using the PAS prediction algorithm for each process, it was revealed that in the radioresistant condition, regulation of the metabolic process, apoptotic process, regulation of programmed cell death, and cell cycle regulation were strongly inhibited (Figure 2C).

**FIGURE 3:**
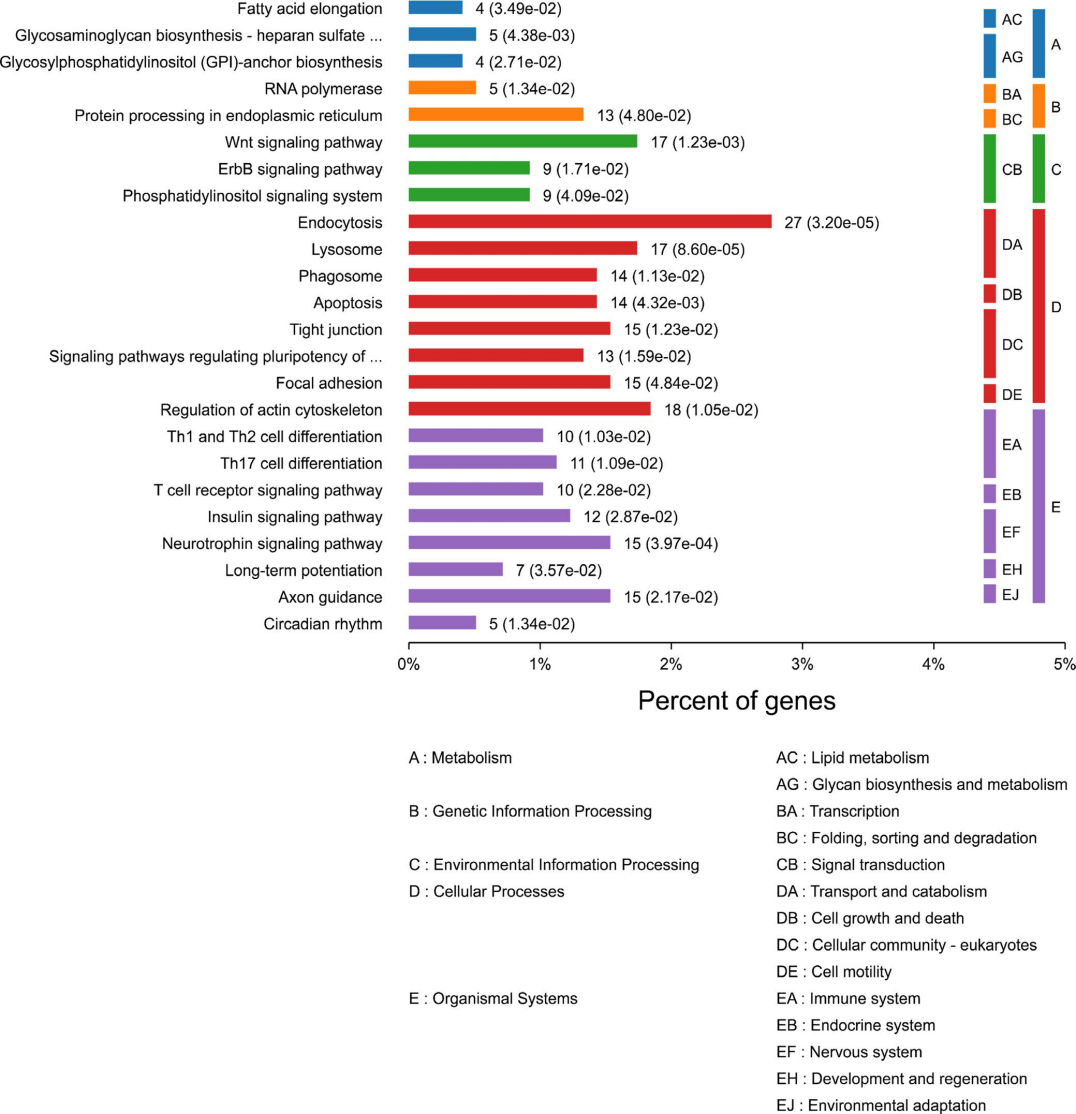
Significantly (*P *< 0.05) enriched KEGG pathways and classification.

Based on the KEGG pathway analysis, three metabolic pathways, including fatty acid elongation, and glycosaminoglycan biosynthesis, were significantly enriched. Moreover, three environment‐related pathways including the Wnt signaling pathway, as well as eight cellular processes including endocytosis, apoptosis, lysosome, and focal adhesion, were enriched. Eight organismal system‐related pathways including T‐cell receptor signaling pathway and neurotrophin signaling pathway were also enriched (Figure 3).

### 3.3 “Hub” sub‐network models for the radioresistant system

**FIGURE 4:**
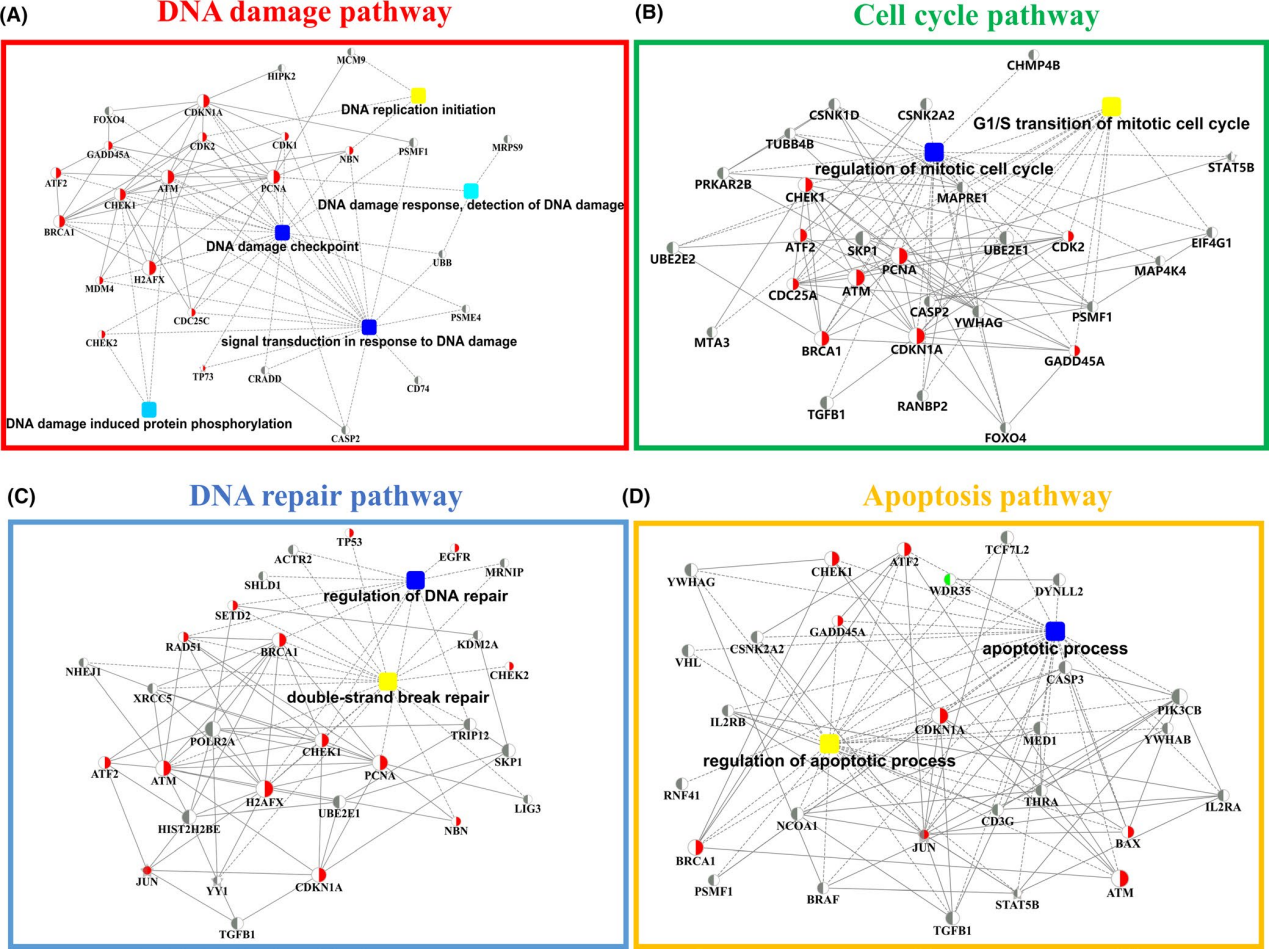
“Hub” sub‐network models related to radioresistance. (A‐D) The main NPC radioresistance models including DNA damage pathway (A), cell cycle pathway (B), DNA repair pathway (C), and apoptosis pathway (D) were constructed by integrating the differentially expressed gene data (left half node of the cycle nodes) with the reported genes (right half node of the cycle nodes). Circle nodes indicate genes, with the right half of the circle colored red representing the gene as a marker gene, the left half colored red representing the gene upregulated in differential expression, and the left half colored green representing the gene downregulated in differential expression. Rectangles indicate KEGG pathways or biological processes. Pathways were colored with gradient color from yellow to blue, with smaller *p* values in yellow and larger *p* values in blue

To find sub‐networks of related genes implicated by multiple forms of biological evidence, we integrated the differentially expressed gene data derived from RNA sequencing, as well as prior knowledge in the form of pathway databases. After integrating the sequenced differential gene data with the reported gene data related to radiosensitivity that we retrieved **(**Table 2), it was found that 10 cancer features, including DNA damage pathway (Figure 4A), cell cycle pathway (Figure 4B), DNA repair pathway (Figure 4C), apoptosis pathway (Figure 4D), stemness pathway (Figure S1A), chromatin pathway (Figure S1B), radiation response pathway (Figure S1C), metal iron response pathway (Figure S1D), epithelial–mesenchymal transition (EMT) pathway (Figure S1E), and cytokine and degranulation pathway (Figure S1F), were enriched. Inspecting each sub‐network through our data revealed several newly discovered common and unique differentially expressed genes related to radioresistance with high connectivity with known genes. It included HIPK2, MCM9 (DNA damage), MAP4K4 (cell cycle), MRNIP (DNA repair), IL2RA (apoptosis), and THBS1 (EMT), implicating several new targets for investigation.

### 3.4 Combination analyses of the differentially expressed genes with GEO data

**FIGURE 5:**
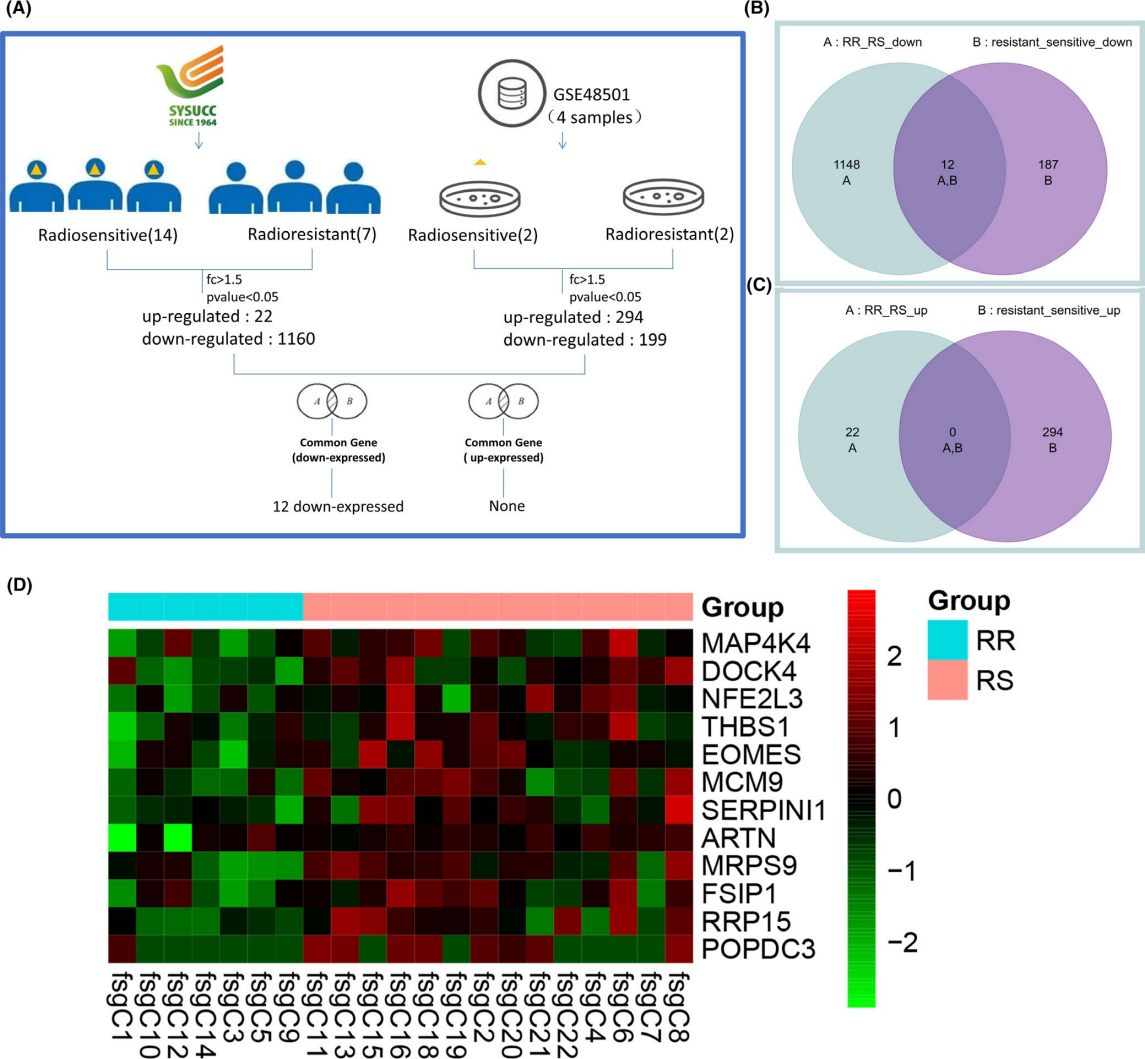
Identification of the overlapping differentially expressed genes between the data of RNA sequencing and the data of GSE48501. RR for radioresistant and RS for radiosensitive. (A) A flowchart of identifying the overlapping differentially expressed genes. (B) A Venn diagram of the overlapping downregulated expressed genes in both the data of RNA sequencing and the data of GSE48501. (C) A Venn diagram of the overlapping upregulated expressed genes in both the data of RNA sequencing and the data of GSE48501. (D) Heatmap of the 12 overlapping differentially expressed genes between the data of RNA sequencing and the data of GSE48501. The horizontal band at the top: cyan: RR, radioresistant group; pink: RS, radiosensitive group. Each row represents a single gene. Green indicates low expression; red indicates high expression

To further search for more reliable genes associated with radioresistance of NPC, we combined the data of RNA sequencing with the GSE48501 data from the GEO database. The data analysis process is shown in Figure 5A. A total of 12 overlapping differentially expressed genes were identified between the two datasets (Figure 5B‐C **)**, including MAP4K4, DOCK4, NFE2L3, THBS1, EOMES, MCM9, SERPINI1, ARTN, MRPS9, FSIP1, RRP15, and POPDC3. These 12 genes were all downregulated in the radioresistant group (Figure 5d).

### 3.5 Survival analysis of the differentially expressed genes

**FIGURE 6:**
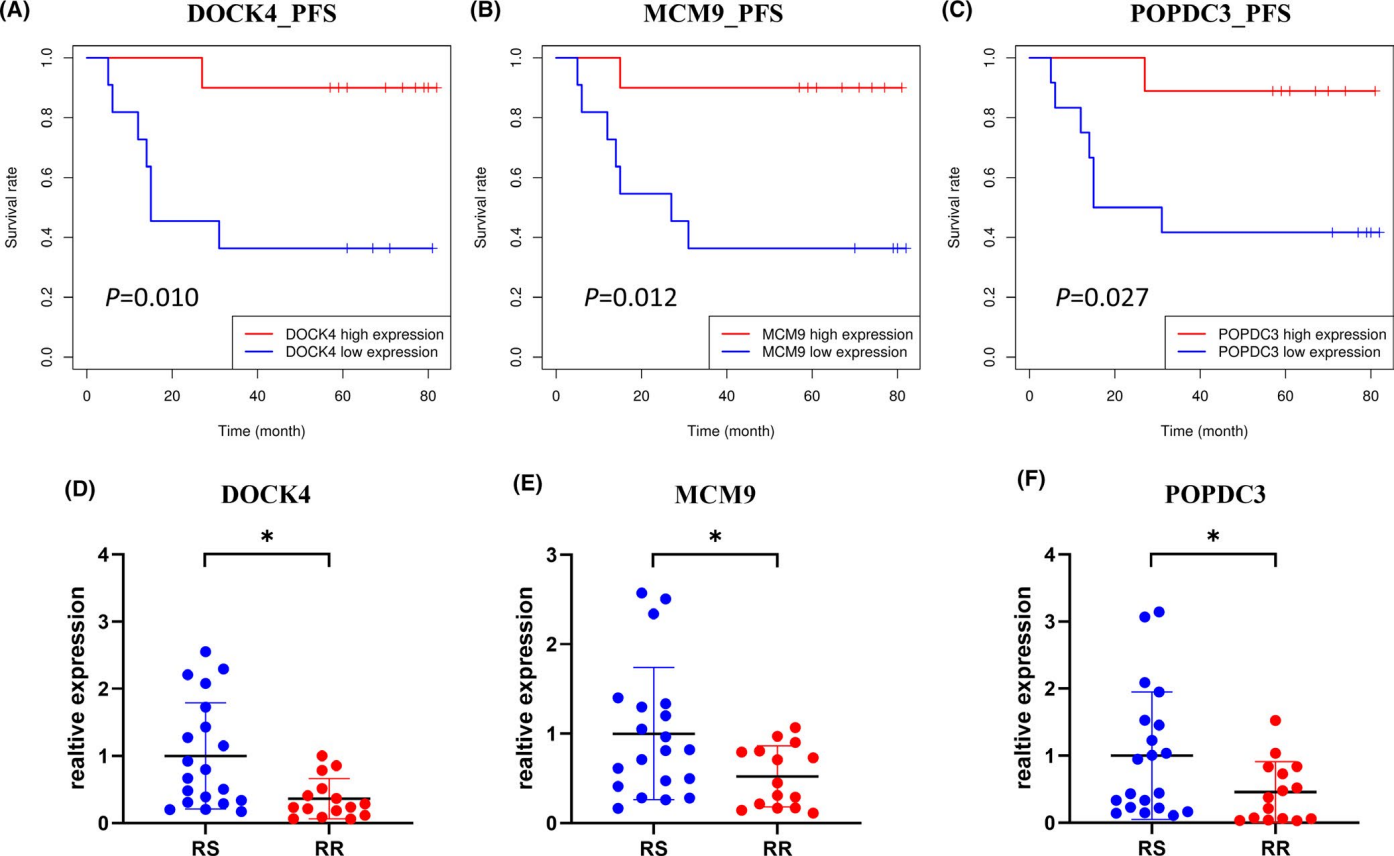
Analysis of the three differentially expressed genes. RR for radioresistant and RS for radiosensitive. (A‐C) Progression‐free survival (PFS) for DOCK4, MCM9, and POPDC3, respectively. The expression value of the gene was divided into two parts: low expression (0%‐50%) and high expression (50%‐100%). (D‐E) Validation of the three differentially expressed genes in 35 NPC biopsy specimens with different radiosensitivity by RT‐qPCR (20 radiosensitive samples and 15 radioresistant samples). Mann–Whitney test was performed to calculate significance. *P < 0.05

To facilitate the understanding of the clinical significance of the differentially expressed genes, survival analyses using the Kaplan–Meier method based on the expression of the genes were conducted. According to the expression levels of the genes in the NPC specimens, the patients were divided into two levels: high expression and low expression. The results of the Kaplan–Meier analyses revealed that patients with a lower level of DOCK4 exhibited significantly shorter PFS (Figure 6A, *P* < 0.05). Similar results were shown in gene MCM9 (Figure 6B), POPDC3 (Figure 6C), ARTN (Figure S2A), MRPS9 (Figure S2B), and SERPIN1 (Figure S2C). In addition, the results of the Kaplan–Meier analyses revealed that the lower expression of ARTN, FSIP1, MCM9, MRPS9 was significantly associated with poorer OS (Figure S2D‐G, *P *< 0.05).

### 3.6 Validation of the differentially expressed genes by RT‐qPCR

RT‐qPCR was performed to validate the expression levels of the differentially expressed genes between 20 radiosensitive NPC specimens and 15 radioresistant NPC specimens. The results of RT‐qPCR revealed that the expression levels of DOCK4 (*P *< 0.05), MCM9 (*P *< 0.05), and POPDC3 (*P *< 0.05) were all significantly downregulated in the radioresistant NPC specimens when compared to those of the radiosensitive NPC specimens (Figure 6D‐F).

## 4 DISCUSSION

At present, radiotherapy resistance has become a major obstacle to the success of NPC treatment. Therefore, increasing attention has been paid to the mechanism of radioresistance in NPC. In recent years, a large number of studies have identified important molecules and biological processes associated with radioresistance by comparing the differences in the expression profiles of genes and other molecules between radiosensitive and radioresistant NPC cell lines.^8^, ^9^, ^10^, ^11^ However, the samples for these studies were all from NPC cell lines cultured in vitro. Few studies have focused on the differences between radioresistant NPC and radiosensitive NPC biopsy tissues so far.

In this study, therefore, we exploited the technology of RNA sequencing to compare the differences in gene expression levels between radioresistant NPC tissues and radiosensitive NPC tissues. As is well known, RNA sequencing, which is considered to be a revolutionary tool for transcriptomics, has high levels of accuracy and reproducibility for detecting gene expression levels.^16^ The results of this study showed that a total of 22 genes were significantly upregulated and 1160 genes were downregulated in the radioresistant group when compared with the genes in the radiosensitive group. With the GO enrichment analysis, we found that the most enriched pathways were concentrated on regulation of the metabolic process, the apoptotic process, regulation of cell cycle, which were consistent with the radioresistance‐related pathways reported in many previous studies.^17^, ^18^, ^19^ These differentially expressed genes were also found to be involved in Notch signaling pathway, response to cytokine, mitotic DNA damage checkpoint, and regulation of DNA repair. Subsequently, the results of the PAS prediction algorithm for each process revealed that under radioresistant conditions, processes including regulation of the metabolic process, apoptotic regulation, and cell cycle regulation were strongly inhibited which might partially explain the underlying mechanism of radioresistance. According to the KEGG pathway analysis, three environment‐related pathways including the Wnt signaling pathway, as well as eight cellular processes including endocytosis, apoptosis, lysosome, and focal adhesion, were enriched. As reported, the Wnt signaling pathway is one of the important pathways that regulate the proliferation, differentiation, and migration of cells,^20^ and dysregulation of the Wnt signaling pathway is closely associated with the development of a variety of tumors such as lung cancer, liver cancer, and breast cancer.^21^ Moreover, three immune‐related pathways, including T‐cell receptor signaling pathway were also enriched, suggesting that the immune status of T cells might be associated with radiation resistance.

By integrating the differentially expressed gene data acquired by RNA sequencing with that of the known genes associated with radiosensitivity reported in the previous literature, we established “hub” sub‐networks of genes related to radioresistance. A total of 10 cancer features, including DNA damage pathway, cell cycle pathway, DNA repair pathway, apoptotic pathway, EMT pathway, chromatin organization pathway, cytokine production and degranulation pathway, stem cell differentiation pathway, and metal iron pathway, were finally enriched. After the subsequent detailed analysis of each pathway, several common participants with high connectivity, such as HIPK2, MCM9, and THBS1, were identified. HIPK2 is considered to be a crucial regulator for targeting apoptosis because it phosphorylates the tumor suppressor p53 in response to DNA damage.^22^, ^23^ It has been shown that HIPK2 knockdown could induce chemoresistance^24^ as well as tumor growth in vivo.^25^ HIPK2 has been reported to be involved in the hypoxic response as a co‐suppressor of hypoxia‐inducible factor‐1α (HIF‐1α), which is a major factor that regulates the transcription of angiogenesis and invasion‐related genes.^26^ THBS1, also known as TSP1, is a member of the thrombospondins (TSPs) family, and its encoded product is a matricellular protein that has the property of limiting angiogenesis by direct effects on endothelial cell migration, proliferation, survival, and apoptosis through CD36, CD47, and integrins.^27^, ^28^ Given its role in delaying angiogenesis, THBS1 has been shown to suppress tumor growth and has also been found to be positively associated with patient survival in several cancers, such as lung,^29^ bladder,^30^ gastric,^31^ and colon cancers.^32^

Furthermore, we combined our RNA sequencing data of radioresistant and radiosensitive NPC tissues with that of radioresistant and radiosensitive NPC cells in the GEO database to find more reliable core genes. A total of 12 overlapping genes were identified finally and the gene expression in three of them including DOCK4, MCM9, and POPDC3 were validated successfully with RT‐qPCR. DOCK4, a member of the dedicator of cytokinesis (DOCK) family, functions as a guanine nucleotide exchange factor (GEF), converting inactive GDP‐bound small GTPases into their active GTP‐bound form and is involved in the regulation of adherens junctions between cells.^33^ DOCK4 has been reported to interact with RAC1,^34^ which is associated with chemoresistance, radioresistance, resistance to targeted therapies, and immune evasion,^35^ implicating a potential promoting mechanism of radioresistance by downregulated DOCK4. A recent study reported that the overexpression of DOCK4 suppresses the tumorigenicity of glioblastomas (GBM) stem‐like cells, and an increased level of DOCK4 predicts improved patient survival of GBM.^36^ Another study showed that DOCK4 expression level is downregulated in paclitaxel‐resistant breast cancers and lncRNA AC073284.4 might sponge miR‐18b‐5p to attenuate the invasion, metastasis, and epithelial–mesenchymal transition of breast cancer cells by upregulating DOCK4 expression.^37^ Similarly, DOCK4 was downregulated in radioresistant NPC in our study. More importantly, the results of the Kaplan–Meier analyses revealed that patients with a lower level of DOCK4 exhibited significantly shorter PFS in our study. MCM9, a member of the mini‐chromosome maintenance (MCM) family, has been shown to play a critical role in DNA replication and repair.^38^ Several studies have found that the complex consisting of MCM9 and its homolog MCM8 can promote homologous recombination‐mediated DNA repair by facilitating RAD51 recruitment to sites of DNA damage and interacting with the MRE11–RAD50–NBS1 complex.^39^, ^40^ In addition, MCM9 has been reported to possess a helicase activity which is required for efficient DNA mismatch repair(MMR), and cells with knockdown of MCM9 exhibit microsatellite instability and MMR deficiency.^41^ Our analysis showed that MCM9 was downregulated in radioresistant NPC, suggesting that the low level of MCM9 might be associated with the radioresistance of NPC. POPDC3, also known as POP3, encodes a transmembrane protein that can facilitate cyclic adenosine monophosphate (cAMP)‐mediated signaling.^42^ Unlike its isoform POPDC1 which acts as a tumor suppressor,^43^ POPDC3 has been found to play distinct roles in different cancer types.^44^ For example, researchers found that knockdown of POPDC3 significantly increased the migration and invasion of gastric cancer cells.^45^ Besides, low expression of POPDC3 was also reported to be associated with metastasis and poor prognosis of gastric cancers.^46^ In contrast, a recent study found that high POPDC3 expression was significantly associated with poor prognosis in head and neck squamous cell carcinoma.^47^ To date, the function of these three genes in NPC has not been reported yet. Therefore, it is necessary to further study the role of these three genes in the radioresistance of NPC in the future.

In conclusion, we analyzed the differentially expressed genes between radioresistant and radiosensitive NPC tissue samples by RNA sequencing and bioinformatics analysis in this study. In addition, 10 major NPC radiation resistance network models were identified through integration analysis with known NPC radiation resistance‐associated genes and mechanisms. Furthermore, we identified three core genes, DOCK4, MCM9, and POPDC3, that may be involved in the radioresistance of NPC. The findings of this study provide new clues for clarifying the mechanism of NPC radioresistance, and further experimental studies of these core genes are warranted.

## CONFLICT OF INTEREST

The authors declare no conflict of interest.

## AUTHOR CONTRIBUTION STATEMENT

F.H performed study concept and design; Z.S, XH.W, and JY.W provided analysis and interpretation of data, and statistical analysis; Z.S, XH.W, JY.W, and J.W performed experimental verification and writing of the paper; RD.H, CY.C, ML.D, and HY.W performed review and revision of the paper; X.L provided technical support. All authors read and approved the final paper.

## ETHICS STATEMENT

The current study was approved by the Clinical Research Ethics Committee of Sun Yat‐sen University Cancer Center and was performed in accordance with the Declaration of Helsinki.

## FUNDING STATEMENT

The authors received no specific funding for this work.

## Supporting information

Figure S1‐S2

## Data Availability

The data that support the findings of this study are available from the corresponding author upon reasonable request.

## References

[1] Chen Y‐P , Chan ATC , Le Q‐T , Blanchard P , Sun Y , Ma J . Nasopharyngeal carcinoma. Lancet. 2019;394(10192):64‐80. 31178151 10.1016/S0140-6736(19)30956-0

[2] Bray F , Ferlay J , Soerjomataram I , Siegel RL , Torre LA , Jemal A . Global cancer statistics 2018: GLOBOCAN estimates of incidence and mortality worldwide for 36 cancers in 185 countries. CA Cancer J Clin. 2018;68(6):394‐424. 30207593 10.3322/caac.21492

[3] Lee AWM , Ng WT , Chan LLK , et al. Evolution of treatment for nasopharyngeal cancer–success and setback in the intensity‐modulated radiotherapy era. Radiother Oncol. 2014;110(3):377‐384. 24630534 10.1016/j.radonc.2014.02.003

[4] Mao Y‐P , Tang L‐L , Chen L , et al. Prognostic factors and failure patterns in non‐metastatic nasopharyngeal carcinoma after intensity‐modulated radiotherapy. Chin J Cancer. 2016;35(1):103. 28031050 10.1186/s40880-016-0167-2 PMC5192583

[5] Ng WT , Lee MCH , Chang ATY , et al. The impact of dosimetric inadequacy on treatment outcome of nasopharyngeal carcinoma with IMRT. Oral Oncol. 2014;50(5):506‐512. 24529762 10.1016/j.oraloncology.2014.01.017

[6] Chan OSH , Sze HCK , Lee MCH , et al. Reirradiation with intensity‐modulated radiotherapy for locally recurrent T3 to T4 nasopharyngeal carcinoma. Head Neck. 2017;39(3):533‐540. 27898191 10.1002/hed.24645

[7] Kong F , Zhou J , Du C , et al. Long‐term survival and late complications of intensity‐modulated radiotherapy for recurrent nasopharyngeal carcinoma. BMC Cancer. 2018;18(1):1139. 30453915 10.1186/s12885-018-5055-5 PMC6245884

[8] Chang J‐C , Chan S‐H , Lin C‐Y , et al. Differentially expressed genes in radioresistant nasopharyngeal cancer cells: gp96 and GDF15. Mol Cancer Ther. 2007;6(8):2271‐2279. 17671084 10.1158/1535-7163.MCT-06-0801

[9] Li X‐H , Qu J‐Q , Yi H , et al. Integrated analysis of differential miRNA and mRNA expression profiles in human radioresistant and radiosensitive nasopharyngeal carcinoma cells. PLoS One. 2014;9(1):e87767. 24498188 10.1371/journal.pone.0087767 PMC3909230

[10] Feng X‐P , Yi H , Li M‐Y , et al. Identification of biomarkers for predicting nasopharyngeal carcinoma response to radiotherapy by proteomics. Cancer Res. 2010;70(9):3450‐3462. 20406978 10.1158/0008-5472.CAN-09-4099

[11] Guo Y , Zhu XD , Qu S , et al. Identification of genes involved in radioresistance of nasopharyngeal carcinoma by integrating gene ontology and protein‐protein interaction networks. Int J Oncol. 2012;40(1):85‐92. 21874234 10.3892/ijo.2011.1172

[12] Ringnér M . What is principal component analysis? Nat Biotechnol. 2008;26(3):303‐304. 18327243 10.1038/nbt0308-303

[13] Borisov NM , Terekhanova NV , Aliper AM , et al. Signaling pathways activation profiles make better markers of cancer than expression of individual genes. Oncotarget. 2014;5(20):10198‐10205. 25415353 10.18632/oncotarget.2548 PMC4259415

[14] Shannon P , Markiel A , Ozier O , et al. Cytoscape: a software environment for integrated models of biomolecular interaction networks. Genome Res. 2003;13(11):2498‐2504. 14597658 10.1101/gr.1239303 PMC403769

[15] Szklarczyk D , Franceschini A , Wyder S , et al. STRING v10: protein–protein interaction networks, integrated over the tree of life. Nucl Acids Res. 2015;43(D1):D447‐D452. 25352553 10.1093/nar/gku1003 PMC4383874

[16] Wang Z , Gerstein M , Snyder M . RNA‐Seq: a revolutionary tool for transcriptomics. Nat Rev Genet. 2009;10(1):57‐63. 19015660 10.1038/nrg2484 PMC2949280

[17] Maity A , McKenna WG , Muschel RJ . The molecular basis for cell cycle delays following ionizing radiation: a review. Radiother Oncol. 1994;31(1):1‐13. 8041894 10.1016/0167-8140(94)90408-1

[18] Igney FH , Krammer PH . Death and anti‐death: tumour resistance to apoptosis. Nat Rev Cancer. 2002;2(4):277‐288. 12001989 10.1038/nrc776

[19] Buckley AM , Lynam‐Lennon N , O'Neill H , O'Sullivan J . Targeting hallmarks of cancer to enhance radiosensitivity in gastrointestinal cancers. Nat Rev Gastroenterol Hepatol. 2020;17(5):298‐313. 32005946 10.1038/s41575-019-0247-2

[20] Steinhart Z , Angers S . Wnt signaling in development and tissue homeostasis. Development. 2018;145(11):dev146589. 29884654 10.1242/dev.146589

[21] Azbazdar Y , Karabicici M , Erdal E , Ozhan G . Regulation of Wnt signaling pathways at the plasma membrane and their misregulation in cancer. Front Cell Dev Biol. 2021;9:631623. 33585487 10.3389/fcell.2021.631623 PMC7873896

[22] Puca R , Nardinocchi L , Givol D , D'Orazi G . Regulation of p53 activity by HIPK2: molecular mechanisms and therapeutical implications in human cancer cells. Oncogene. 2010;29(31):4378‐4387. 20514025 10.1038/onc.2010.183

[23] Hofmann TG , Möller A , Sirma H , et al. Regulation of p53 activity by its interaction with homeodomain‐interacting protein kinase‐2. Nat Cell Biol. 2002;4(1):1‐10. 11740489 10.1038/ncb715

[24] Di Stefano V , Rinaldo C , Sacchi A , Soddu S , D'Orazi G . Homeodomain‐interacting protein kinase‐2 activity and p53 phosphorylation are critical events for cisplatin‐mediated apoptosis. Exp Cell Res. 2004;293(2):311‐320. 14729469 10.1016/j.yexcr.2003.09.032

[25] D'Orazi G , Sciulli MG , Di Stefano V , et al. Homeodomain‐interacting protein kinase‐2 restrains cytosolic phospholipase A2‐dependent prostaglandin E2 generation in human colorectal cancer cells. Clin Cancer Res. 2006;12(3 Pt 1):735‐741. 16467083 10.1158/1078-0432.CCR-05-1557

[26] Nardinocchi L , Puca R , Givol D , D'Orazi G . HIPK2‐a therapeutical target to be (re)activated for tumor suppression: role in p53 activation and HIF‐1α inhibition. Cell Cycle. 2010;9(7):1270‐1275. 20234185 10.4161/cc.9.7.11125

[27] Zhao C , Isenberg JS , Popel AS . Human expression patterns: qualitative and quantitative analysis of thrombospondin‐1 under physiological and pathological conditions. J Cell Mol Med. 2018;22(4):2086‐2097. 29441713 10.1111/jcmm.13565 PMC5867078

[28] Lawler PR , Lawler J . Molecular basis for the regulation of angiogenesis by thrombospondin‐1 and ‐2. Cold Spring Harb Perspect Med. 2012;2(5):a006627. 22553494 10.1101/cshperspect.a006627 PMC3331684

[29] Papadaki C , Mavroudis D , Trypaki M , et al. Tumoral expression of TXR1 and TSP1 predicts overall survival of patients with lung adenocarcinoma treated with first‐line docetaxel‐gemcitabine regimen. Clin Cancer Res. 2009;15(11):3827‐3833. 19435835 10.1158/1078-0432.CCR-08-3027

[30] Grossfeld GD , Ginsberg DA , Stein JP , et al. Thrombospondin‐1 expression in bladder cancer: association with p53 alterations, tumor angiogenesis, and tumor progression. J Natl Cancer Inst. 1997;89(3):219‐227. 9017002 10.1093/jnci/89.3.219

[31] Nakao T , Kurita N , Komatsu M , et al. Expression of thrombospondin‐1 and Ski are prognostic factors in advanced gastric cancer. Int J Clin Oncol. 2011;16(2):145‐152. 21107877 10.1007/s10147-010-0147-5

[32] Kaio E , Tanaka S , Oka S , et al. Clinical significance of thrombospondin‐1 expression in relation to vascular endothelial growth factor and interleukin‐10 expression at the deepest invasive tumor site of advanced colorectal carcinoma. Int J Oncol. 2003;23(4):901‐911. 12963968

[33] Upadhyay G , Goessling W , North TE , Xavier R , Zon LI , Yajnik V . Molecular association between beta‐catenin degradation complex and Rac guanine exchange factor DOCK4 is essential for Wnt/beta‐catenin signaling. Oncogene. 2008;27(44):5845‐5855. 18641688 10.1038/onc.2008.202 PMC4774646

[34] Gadea G , Blangy A . Dock‐family exchange factors in cell migration and disease. Eur J Cell Biol. 2014;93(10–12):466‐477. 25022758 10.1016/j.ejcb.2014.06.003

[35] Cardama GA , Alonso DF , Gonzalez N , et al. Relevance of small GTPase Rac1 pathway in drug and radio‐resistance mechanisms: opportunities in cancer therapeutics. Crit Rev Oncol Hematol. 2018;124:29‐36. 29548483 10.1016/j.critrevonc.2018.01.012

[36] Debruyne DN , Turchi L , Burel‐Vandenbos F , et al. DOCK4 promotes loss of proliferation in glioblastoma progenitor cells through nuclear beta‐catenin accumulation and subsequent miR‐302‐367 cluster expression. Oncogene. 2018;37(2):241‐254. 28925399 10.1038/onc.2017.323

[37] Wang Y‐Y , Yan L , Yang S , et al. Long noncoding RNA AC073284.4 suppresses epithelial‐mesenchymal transition by sponging miR‐18b‐5p in paclitaxel‐resistant breast cancer cells. J Cell Physiol. 2019;234(12):23202‐23215. 31215650 10.1002/jcp.28887

[38] Lutzmann M , Méchali M . MCM9 binds Cdt1 and is required for the assembly of prereplication complexes. Mol Cell. 2008;31(2):190‐200. 18657502 10.1016/j.molcel.2008.07.001

[39] Park J , Long DT , Lee KY , et al. The MCM8‐MCM9 complex promotes RAD51 recruitment at DNA damage sites to facilitate homologous recombination. Mol Cell Biol. 2013;33(8):1632‐1644. 23401855 10.1128/MCB.01503-12 PMC3624244

[40] Lee KY , Im J‐S , Shibata E , et al. MCM8‐9 complex promotes resection of double‐strand break ends by MRE11‐RAD50‐NBS1 complex. Nat Commun. 2015;6:7744. 26215093 10.1038/ncomms8744 PMC4525285

[41] Traver S , Coulombe P , Peiffer I , et al. MCM9 is required for mammalian DNA mismatch repair. Mol Cell. 2015;59(5):831‐839. 26300262 10.1016/j.molcel.2015.07.010

[42] Brand T , Schindler R . New kids on the block: the Popeye domain containing (POPDC) protein family acting as a novel class of cAMP effector proteins in striated muscle. Cell Signal. 2017;40:156‐165. 28939104 10.1016/j.cellsig.2017.09.015 PMC6562197

[43] Han P , Lei Y , Li D , Liu J , Yan W , Tian D . Ten years of research on the role of BVES/ POPDC1 in human disease: a review. Onco Targets Ther. 2019;12:1279‐1291. 30863095 10.2147/OTT.S192364 PMC6388986

[44] Amunjela JN , Swan AH , Brand T . The role of the popeye domain containing gene family in organ homeostasis. Cells. 2019;8(12):1594. 10.3390/cells8121594 PMC6952887 31817925

[45] Kim M , Jang H‐R , Haam K , et al. Frequent silencing of popeye domain‐containing genes, BVES and POPDC3, is associated with promoter hypermethylation in gastric cancer. Carcinogenesis. 2010;31(9):1685‐1693. 20627872 10.1093/carcin/bgq144

[46] Luo D , Lu ML , Zhao GF , et al. Reduced Popdc3 expression correlates with high risk and poor survival in patients with gastric cancer. World J Gastroenterol. 2012;18(19):2423‐2429. 22654436 10.3748/wjg.v18.i19.2423 PMC3353379

[47] He XU , Xu H , Zhao W , et al. POPDC3 is a potential biomarker for prognosis and radioresistance in patients with head and neck squamous cell carcinoma. Oncol Lett. 2019;18(5):5468‐5480. 31612055 10.3892/ol.2019.10888 PMC6781657

[48] Bian L , Meng Y , Zhang M , Li D . MRE11‐RAD50‐NBS1 complex alterations and DNA damage response: implications for cancer treatment. Mol Cancer. 2019;18(1):169. 31767017 10.1186/s12943-019-1100-5 PMC6878665

[49] Scully R , Panday A , Elango R , Willis NA . DNA double‐strand break repair‐pathway choice in somatic mammalian cells. Nat Rev Mol Cell Biol. 2019;20(11):698‐714. 31263220 10.1038/s41580-019-0152-0 PMC7315405

[50] He X , Fan S . hsa‐miR‐212 modulates the radiosensitivity of glioma cells by targeting BRCA1. Oncol Rep. 2018;39(3):977‐984. 29286157 10.3892/or.2017.6156 PMC5802039

[51] Weterings E , Chen DJ . The endless tale of non‐homologous end‐joining. Cell Res. 2008;18(1):114‐124. 18166980 10.1038/cr.2008.3

[52] Celeste A , Fernandez‐Capetillo O , Kruhlak MJ , et al. Histone H2AX phosphorylation is dispensable for the initial recognition of DNA breaks. Nat Cell Biol. 2003;5(7):675‐679. 12792649 10.1038/ncb1004

[53] Eischen CM . Role of Mdm2 and Mdmx in DNA repair. J Mol Cell Biol. 2017;9(1):69‐73. 27932484 10.1093/jmcb/mjw052 PMC5439402

[54] Stewart GS , Wang B , Bignell CR , Taylor AM , Elledge SJ . MDC1 is a mediator of the mammalian DNA damage checkpoint. Nature. 2003;421(6926):961‐966. 12607005 10.1038/nature01446

[55] Gou Q , Xie Y , Liu L , et al. Downregulation of MDC1 and 53BP1 by short hairpin RNA enhances radiosensitivity in laryngeal carcinoma cells. Oncol Rep. 2015;34(1):251‐257. 25976740 10.3892/or.2015.3980

[56] Cuella‐Martin R , Oliveira C , Lockstone HE , Snellenberg S , Grolmusova N , Chapman JR . 53BP1 integrates DNA repair and p53‐dependent cell fate decisions via distinct mechanisms. Mol Cell. 2016;64(1):51‐64. 27546791 10.1016/j.molcel.2016.08.002 PMC5065530

[57] Sunavala‐Dossabhoy G , De Benedetti A . Tousled homolog, TLK1, binds and phosphorylates Rad9; TLK1 acts as a molecular chaperone in DNA repair. DNA Repair (Amst). 2009;8(1):87‐102. 18940270 10.1016/j.dnarep.2008.09.005

[58] Bhoumik A , Takahashi S , Breitweiser W , Shiloh Y , Jones N , Ronai Z . ATM‐dependent phosphorylation of ATF2 is required for the DNA damage response. Mol Cell. 2005;18(5):577‐587. 15916964 10.1016/j.molcel.2005.04.015 PMC2954254

[59] Schär P , Fäsi M , Jessberger R . SMC1 coordinates DNA double‐strand break repair pathways. Nucleic Acids Res. 2004;32(13):3921‐3929. 15280507 10.1093/nar/gkh716 PMC506803

[60] Smith J , Tho LM , Xu N , Gillespie DA . The ATM‐Chk2 and ATR‐Chk1 pathways in DNA damage signaling and cancer. Adv Cancer Res. 2010;108:73‐112. 21034966 10.1016/B978-0-12-380888-2.00003-0

[61] Reinhardt HC , Yaffe MB . Kinases that control the cell cycle in response to DNA damage: Chk1, Chk2, and MK2. Curr Opin Cell Biol. 2009;21(2):245‐255. 19230643 10.1016/j.ceb.2009.01.018 PMC2699687

[62] Sørensen CS , Syljuåsen RG , Falck J , et al. Chk1 regulates the S phase checkpoint by coupling the physiological turnover and ionizing radiation‐induced accelerated proteolysis of Cdc25A. Cancer Cell. 2003;3(3):247‐258. 12676583 10.1016/s1535-6108(03)00048-5

[63] Kaneko Y , Watanabe N , Morisaki H , et al. Cell‐cycle‐dependent and ATM‐independent expression of human Chk1 kinase. Oncogene. 1999;18(25):3673‐3681. 10391675 10.1038/sj.onc.1202706

[64] Wang YA , Elson A , Leder P . Loss of p21 increases sensitivity to ionizing radiation and delays the onset of lymphoma in atm‐deficient mice. Proc Natl Acad Sci U S A. 1997;94(26):14590‐14595. 9405657 10.1073/pnas.94.26.14590 PMC25064

[65] King TC , Estalilla OC , Safran H . Role of p53 and p16 gene alterations in determining response to concurrent paclitaxel and radiation in solid tumor. Semin Radiat Oncol. 1999;9(2 Suppl 1):4‐11. 10210535

[66] Hollander MC , Fornace AJ Jr . Genomic instability, centrosome amplification, cell cycle checkpoints and Gadd45a. Oncogene. 2002;21(40):6228‐6233. 12214253 10.1038/sj.onc.1205774

[67] Liu X , Tu Y , Wang Y , et al. Reversible inhibitor of CRM1 sensitizes glioblastoma cells to radiation by blocking the NF‐κB signaling pathway. Cancer Cell Int. 2020;20:97. 32256206 10.1186/s12935-020-01186-y PMC7106748

[68] Nakanishi K , Taniguchi T , Ranganathan V , et al. Interaction of FANCD2 and NBS1 in the DNA damage response. Nat Cell Biol. 2002;4(12):913‐920. 12447395 10.1038/ncb879

[69] Gudkov AV , Komarova EA . The role of p53 in determining sensitivity to radiotherapy. Nat Rev Cancer. 2003;3(2):117‐129. 12563311 10.1038/nrc992

[70] El‐Deiry WS . The role of p53 in chemosensitivity and radiosensitivity. Oncogene. 2003;22(47):7486‐7495. 14576853 10.1038/sj.onc.1206949

[71] Mohamed MS , Bishr MK , Almutairi FM , Ali AG . Inhibitors of apoptosis: clinical implications in cancer. Apoptosis. 2017;22(12):1487‐1509. 29067538 10.1007/s10495-017-1429-4

[72] Holley AK , Xu Y , St Clair DK , St Clair WH . RelB regulates manganese superoxide dismutase gene and resistance to ionizing radiation of prostate cancer cells. Ann N Y Acad Sci. 2010;1201:129‐136. 20649549 10.1111/j.1749-6632.2010.05613.x PMC3107504

[73] D'Auria F , Centurione L , Centurione MA , Angelini A , Di Pietro R . Regulation of cancer cell responsiveness to ionizing radiation treatment by cyclic AMP response element binding nuclear transcription factor. Front Oncol. 2017;7:76. 28529924 10.3389/fonc.2017.00076 PMC5418225

[74] Verheij M , van Blitterswijk WJ , Bartelink H . Radiation‐induced apoptosis–the ceramide‐SAPK signaling pathway and clinical aspects. Acta Oncol. 1998;37(6):575‐581. 9860316 10.1080/028418698430287

[75] Macedo‐Silva C , Miranda‐Gonçalves V , Henrique R , Jerónimo C , Bravo I . The critical role of hypoxic microenvironment and epigenetic deregulation in esophageal cancer radioresistance. Genes (Basel). 2019;10(11):927. 10.3390/genes10110927 PMC6896142 31739546

[76] Moeller BJ , Dewhirst MW . HIF‐1 and tumour radiosensitivity. Br J Cancer. 2006;95(1):1‐5. 16735998 10.1038/sj.bjc.6603201 PMC2360497

[77] Wilson WR , Hay MP . Targeting hypoxia in cancer therapy. Nat Rev Cancer. 2011;11(6):393‐410. 21606941 10.1038/nrc3064

[78] Solberg TD , Nearman J , Mullins J , Li S , Baranowska‐Kortylewicz J . Correlation between tumor growth delay and expression of cancer and host VEGF, VEGFR2, and osteopontin in response to radiotherapy. Int J Radiat Oncol Biol Phys. 2008;72(3):918‐926. 19014781 10.1016/j.ijrobp.2008.06.1925 PMC2585106

