# Supplementary material for: Key radioresistance regulation models and marker genes identified by integrated transcriptome analysis in nasopharyngeal carcinoma
Source: Cancer Med. 2021 Aug 25;10(20):7404–17. doi: 10.1002/cam4.4228 (PMC8525106; doi:10.1002/cam4.4228)
Supplement: Supplementary file 1 — Figure S1‐S2 [file CAM4-10-7404-s001.docx]

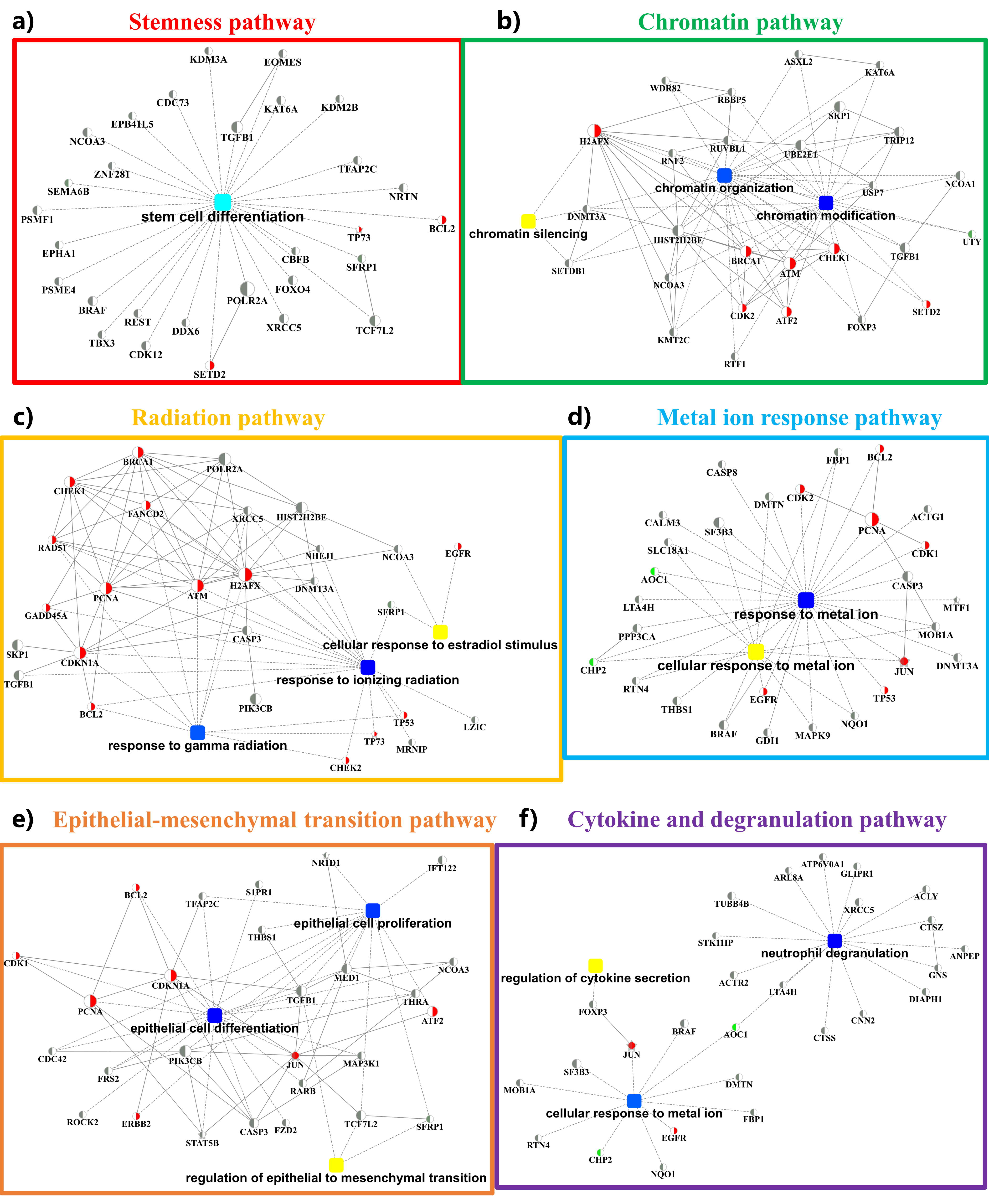


**Figure S1.** “Hub” sub-network models related to radioresistance.

a)-f) Main NPC radioresistance models including Stemness pathway (a), Chromatin pathway (b), Radiation pathway (c), Metal ion response pathway (d), epithelial-mesenchymal transition (EMT) pathway (e), and cytokine and degranulation pathway (f). Circle nodes indicate genes, with the right half of the circle colored red representing the gene as a marker gene, the left half colored red representing the gene up-regulated in differential expression, and the left half colored green representing the gene down-regulated in differential expression. Rectangles indicate KEGG pathways or biological processes. Pathways were colored with gradient color from yellow to blue, with smaller *P* values in yellow and larger *P* values in blue.


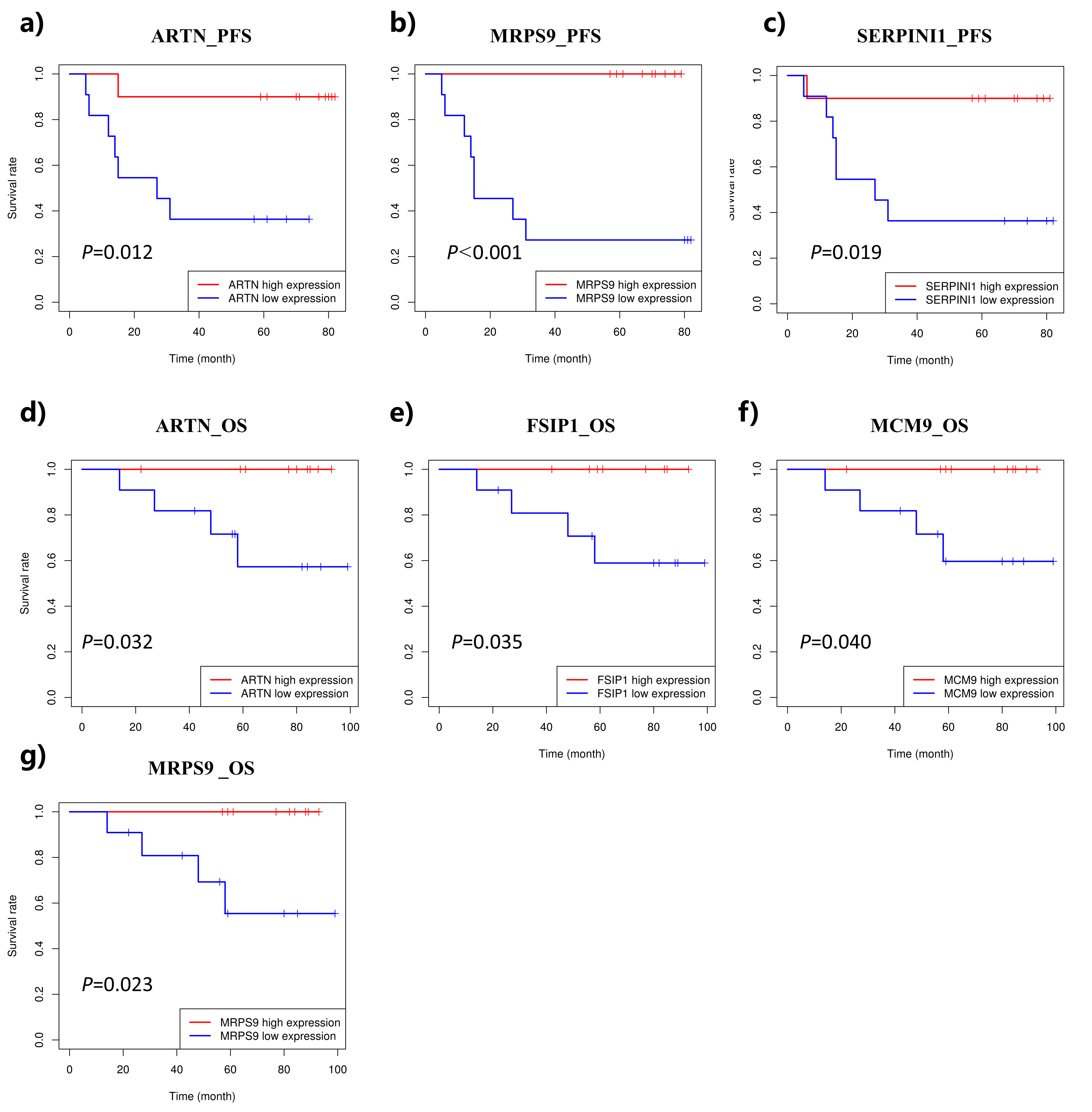


**Figure S2.** Survival Analysis of the differentially expressed genes.

a)-c) Progression-free survival (PFS) for ARTN, MRPS9, and SERPINI1, respectively.

d)-g) Overall survival (OS) for ARTN, FSIP1, MCM9, MRPS9, respectively. *P* values < 0.05 were considered statistically significant.
